# Advanced Glycation End-Products Can Activate or Block Bitter Taste Receptors

**DOI:** 10.3390/nu11061317

**Published:** 2019-06-12

**Authors:** Appalaraju Jaggupilli, Ryan Howard, Rotimi E. Aluko, Prashen Chelikani

**Affiliations:** 1 Manitoba Chemosensory Biology Research Group, Department of Oral Biology, University of Manitoba, Children’s Hospital Research Institute of Manitoba (CHRIM), Winnipeg, MB R3E 0W4, Canada; Appalaraju.Jaggupilli@umanitoba.ca (A.J.); umhowarr@myumanitoba.ca (R.H.); 2 Department of Food and Human Nutritional Sciences, University of Manitoba, Winnipeg, MB R3T 2N2, Canada; Rotimi.Aluko@umanitoba.ca

**Keywords:** bitter taste receptor, advanced glycation end-products, bitter blockers, GPCR, calcium mobilization

## Abstract

Bitter taste receptors (T2Rs) are expressed in several tissues of the body and are involved in a variety of roles apart from bitter taste perception. Advanced glycation end-products (AGEs) are produced by glycation of amino acids in proteins. There are varying sources of AGEs, including dietary food products, as well as endogenous reactions within our body. Whether these AGEs are T2R ligands remains to be characterized. In this study, we selected two AGEs, namely, glyoxal-derived lysine dimer (GOLD) and carboxymethyllysine (CML), based on their predicted interaction with the well-studied T2R4, and its physiochemical properties. Results showed predicted binding affinities (*K_d_*) for GOLD and CML towards T2R4 in the nM and μM range, respectively. Calcium mobilization assays showed that GOLD inhibited quinine activation of T2R4 with IC_50_ 10.52 ± 4.7 μM, whilst CML was less effective with IC_50_ 32.62 ± 9.5 μM. To characterize whether this antagonism was specific to quinine activated T2R4 or applicable to other T2Rs, we selected T2R14 and T2R20, which are expressed at significant levels in different human tissues. A similar effect of GOLD was observed with T2R14; and in contrast, GOLD and CML activated T2R20 with an EC_50_ of 79.35 ± 29.16 μM and 65.31 ± 17.79 μM, respectively. In this study, we identified AGEs as novel T2R ligands that caused either activation or inhibition of different T2Rs.

## 1. Introduction

Bitter taste receptors (T2Rs) are generally recognized for their role in perceiving bitter taste, which is one of the five basic taste modalities in mammals [1,2]. Humans express 25 T2R subtypes that belong to the G protein-coupled receptor superfamily [3]. Besides their role in bitter taste perception, extensive literature has shown that T2Rs are expressed in several other extra-oral tissues and they perform diverse chemosensory functions depending on the tissue of origin [4].

Information on the endogenous ligands for T2Rs is poorly understood. With the characterization of T2R signaling in extra-oral tissues, it is important to identify possible endogenous ligands that regulate the functions of T2Rs. There are 1041 compounds currently known to activate the T2Rs [5]. Amongst these, over 800 ligand-receptor associations were identified and nearly 260 compounds are associated with at least one T2R [5]. However, when compared to bitter agonists, the number of characterized bitter blockers are substantially lower [6,7,8]. The bitter agonists include many plant products, such as alkaloids, terpenoids, flavonoids, phenols, glycosides, and synthetic compounds like dextromethorphan [9]. Although at the industry level, a number of approaches have been used to mask the bitter taste of pharmaceutical products, the dearth of specific T2R blockers still remains [6,10]. Previously, we summarized the experimentally characterized bitter blockers and industrially used bitter maskers [6]. A recent study showed that the non-caloric sweeteners saccharin and cyclamate inhibited each other’s activation of their respective T2Rs [11]. However, none of the characterized blockers could inhibit all the 25 T2Rs.

Previously, we have also reported *N*α, *N*α-bis(carboxymethyl)-l-lysine (BCML) as a potential competitive inhibitor of T2R4 [12]. BCML is an amino acid derivate of lysine that is analogous to an advanced glycation end-product (AGE), Nε-carboxymethyl lysine (CML). AGEs are a spectrum of glycated heterogeneous compounds formed by non-enzymatic reactions between reducing sugars (α-carbonylic compounds) and amino group containing residues (lysine and/or arginine) in proteins through a process termed non-enzymatic browning or the Maillard reaction [13,14,15]. AGEs are characterized as non-crosslinking and non-fluorescent (CML, CMhL, Pyrralline, Imidazolone); crosslinking and non-fluorescent (carboxyethyl lysine, glyoxal-derived lysine dimer, methyl glyoxal-derived lysine dimer, 3-deoxyglucosone-derived lysine dimer, and glucosepane); and crosslinking and fluorescent (Pentosidine. Argpyrimidine and Crossline) [16,17].

AGEs occur from both exogenous and endogenous reactions. The sources for exogenous AGEs are tobacco smoke, dietary food products, and uncooked animal-derived food products [18,19]. Furthermore, prolonged cooking or thermal processing can generate AGEs through oxidation of intermediate metabolites, such as glyoxal, methyl glyoxal, and 3-deoxyglucosone [15]. The AGEs give a distinctive aroma and taste in thermally processed foods. In normal physiological conditions, AGEs are synthesized from the absorbed reducing sugars found in the blood [20]. The AGEs can chemically modify proteins, lipids, and nucleic acids [15,19]. These AGE-modified molecules are further recognized by the endogenous secretory receptors or scavenger receptors on macrophages [21]. Subsequently, the molecules are degraded and excreted through the urinary system to limit toxic effects [22]. However, in pathological conditions, such systems may lead to cellular dysfunctions due to the accumulation of AGEs [21]. Consequently, this causes vascular diseases like atherosclerosis, diabetes, and chronic kidney disease [13,23,24]. Excessive generation of AGEs is very harmful and causes tissue damage through the formation of covalent crosslinks with intracellular and extracellular matrix proteins like collagen [13,25]. These crosslinks increase tissue stiffness and lead to organ dysfunction [21,22,26]. However, on a positive note, restriction in dietary AGEs showed an improvement in wound healing, insulin resistance, and cardiovascular diseases [27]. It has also been shown that their restriction improved the lifespan of animal models [28]. Interactions of AGEs with the receptor for advanced glycated end-products (RAGE) and other receptors, such as AGE-R1, AGE-R2, and AGE-R3, accelerate oxidative stress by forming reactive oxygen species (ROS) [21]. However, cell surface receptors other than RAGE are not well characterized for AGEs. Recently, we showed that enzymatic meat protein hydrolysates can interact with T2R4 expressed in a heterologous system [29,30]. Fractions from different protein hydrolysates either activated or blocked calcium mobilization. AGEs being amino acid substituents may interact with T2Rs.

Therefore, considering the above studies and previously reported BCML as a T2R4 blocker, the aim of this study was to investigate whether AGEs were novel ligands for T2Rs. We chose several (21) AGEs from the Maillard’s reaction products list available at the International Maillard’s Reaction Society (IMARS) website, and they were used for molecular docking analysis with T2R4. Based on their scoring functions, commercial availability, and solubility in our calcium assay buffer, we selected two AGEs (GOLD and CML) for further studies. These compounds were tested on T2R4 in cell based calcium mobilization assays. Next, these compounds were tested for their activity with T2R14 and T2R20. The rationale for selecting these T2Rs was based on their significant expression levels in human tissues, structure-function information, and agonist diversity. Others and we have previously shown that T2R4 is moderately expressed whilst T2R14 and T2R20 are highly expressed in several extra-oral tissues [31,32,33,34,35,36,37]. T2R4 and T2R14 are well characterized, where the latter is a broadly tuned T2R for several ligands [9,38,39,40]. Despite its high expression level, the known ligands for T2R20 are relatively few [9,41,42]. The results suggest AGEs as novel T2R ligands that cause either the activation or inhibition of the T2Rs.

## 2. Materials and Methods

### 2.1. Chemicals

The cell-culture medium was composed of Dulbecco’s modified Eagle’s medium-F12 (Gibco, Life technologies, Carlsbad, CA, USA) supplemented with 10% heat-inactivated fetal bovine serum (Sigma, Burlington, MA, USA) and 1% penicillin-streptomycin (Gibco, Life technologies). We used hygromycin (Sigma Aldrich, Oakville, ON, Canada) in the selection medium to generate stable cells. All bitter compounds (quinine hydrochloride, diphenhydramine or DPH, and cromolyn) were purchased from Sigma Aldrich (Oakville, ON, Canada). We purchased glyoxal-derived lysine dimer (GOLD) and Nε-Carboxymethyl-l-lysine (CML) from PolyPeptide Laboratories (Strasbourg, France). All the compounds were freshly prepared using calcium assay buffer (1X HBSS, 20 mM HEPES) on the day of the experiment. Calcium sensitive dye Fluo-4 NW kit was purchased from Life technologies (Carlsbad, CA USA).

### 2.2. Molecular Biology and Cell Culture

Human Embryonic Kidney (HEK293T) cell line was purchased from ATCC (Manassas, VA, USA). N-terminal FLAG epitope tagged human TAS2R4, TAS2R14, and TAS2R20 genes were codon-optimized for expression in mammalian cells and cloned into the KpnI-NotI site of pcDNA 3.1/Hygro (+) expression vector, and they were commercially synthesized (GenScript Inc., Piscataway, MA, USA) as previously described in Reference [43]. We generated stable HEK293T cells expressing the above T2Rs in the selection medium containing 200 μg/mL hygromycin as described previously in References [43,44,45].

### 2.3. Functional Assays

Calcium mobilization assays on each T2R expressing stable cells were performed by incubation with Fluo-4 NW dye containing probenecid (2.5 mM) for 35 min at 37 °C, followed by 35 min at room temperature. We then treated the cells either with the putative agonist alone or in combination with the AGE compound. To elucidate the action of AGEs on T2Rs, the cells were initially treated with a fixed concentration (100 μM). To determine IC_50_ for AGE compounds, the agonist concentration was kept constant whilst the AGE compound was added in a concentration dependent manner (3 μM to 200 μM). To determine EC_50_ for the agonists, we used a concentration range from 3 μM to 200 μM. The Flexstation-3 automated fluorescence microplate reader (Molecular devices) was used to measure the calcium at 525 nm emission, followed by excitation at 494 nm. ΔRFU (relative fluorescence units) were calculated by the system based on the difference between the maximum response and minimum response. We used HEK293T cells as the control for baseline subtraction. The data were collected from at least three independent experiments performed in triplicates and analyzed using GraphPad Prism V. 6.0 (GraphPadSoftware, La Jolla, CA, USA).

### 2.4. Molecular Modeling and Ligand Docking

Modeling and docking protocols were performed as described previously in References [12,46]. Briefly, an experimentally validated T2R4 model was prepared for ligand docking using the Discovery Studio modeling suite (DS 4.0) (Dassault Systemes BIOVIA, San Diego, CA, USA) [12,41]. From the International Maillard Reaction Society (IMARS), we listed and identified the AGEs in public domains, such as PubChem, ChEBI, and ChemSpider. For the derivatives of AGEs where the identifiers were not available, their structures were manually drawn using the Biovia ChemDraw software (v. 16.1) (Dassault Systemes BIOVIA, San Diego, CA, USA) to generate the 3D structures. Subsequently, all the 3D structures were prepared for molecular docking using the “Prepare Ligands” protocol available in DS 4.0. This protocol changes ionization, generates tautomers and isomers, fixes bad valences, and generates 3D coordinates. These were energy minimized with the CHARMM program using a smart minimizer algorithm with 1000 steps at a gradient tolerance of 0.01. Furthermore, the structures were filtered using the Lipinski rule of five and the Veber rule based on default cut-off values for hydrogen bond donors and acceptors, molecular weight, AlogP, rotatable bonds, and the polar surface area. [47] All the compounds passed through the filtering and further assessed for drug like properties. This was done using the ADMET descriptor (absorption, distribution, metabolism, and excretion) and TOPKAT (Toxicity Prediction by Komputer Assisted Technology) protocol [48]. The CDOCKER program was used to dock these compounds into the T2R4 model [49]. The coordinates for the input site sphere were taken as *X* = 59.45, *Y* = 67.28, and *Z* = 54.55, with a radius of 14 Å. We generated 20 refined poses with the top 10 hits for each ligand leaving default parameters for random conformations and orientations to refine. The docked compounds were subjected to in-situ ligand minimization, and 11 different scoring functions (LigScore1_Dreiding, LigScore2_Drieding, PLP1, PLP2 Jain, PMF, PMF04, CDocker_Energy, Ludi1, Ludi2 and Ludi3) were run, where each function had its own individual algorithm calculations [46]. Ludi3 scores were used to calculate the predicted binding affinity using the formula: Ludi3 score = −100 log Kd as described earlier in Reference [50]. PyMol visualizer was used to generate the publication quality images [51].

### 2.5. Statistical Analysis

We performed all the statistical analyses using GraphPad Prism version v6.0 (Graphpad software, La Jolla, CA, USA). Statistical analysis was performed using one-way analysis of variance (ANOVA) with a Tukey’s multiple comparison post hoc test, from a minimum of 3–4 independent experiments to determine the statistical significance (* *p* < 0.05, ** *p* < 0.01) wherever applicable.

## 3. Results

### 3.1. Prediction of Binding Affinities of AGEs for T2R4

Previous studies on amino acid derivatives from our lab reported BCML as an antagonist of T2R4 with an IC_50_ value of 60 nM against quinine [12]. An antagonist is a compound that blocks the biological activity of a receptor. As mentioned before, we selected 21 AGE compounds. For the docking experiments, T2R4 was selected because its ligand-binding pocket and interactions with BCML were previously reported, and thus it could act as a good reference model [12]. All the selected AGE compounds were docked into the same binding region [12,41]. Quinine, BCML, and γ-aminobutyric acid (GABA) were included as control compounds. Ludi scoring functions (Ludi3) were used to calculate the binding affinities (*K_d_*) to rank the docked poses as described in the methods section (Table 1). Based on this ranking, we examined the top 10 compounds for commercial availability and solubility in our calcium assay buffer. For example, pentosidine and Nε-carboxyethyl lysine were available commercially, but were insoluble or only slightly soluble in our calcium assay buffer. Subsequently, we selected the commercially available compounds, Glyoxal-derived lysine dimer (GOLD) and CML, for further testing of their effect on T2Rs. The predicted binding affinities (*K_d_*) were 0.1096 μM for GOLD and 0.0457 μM for CML (Table 1)

### 3.2. GOLD and CML Inhibit Activation of T2R4

**Figure 1:**
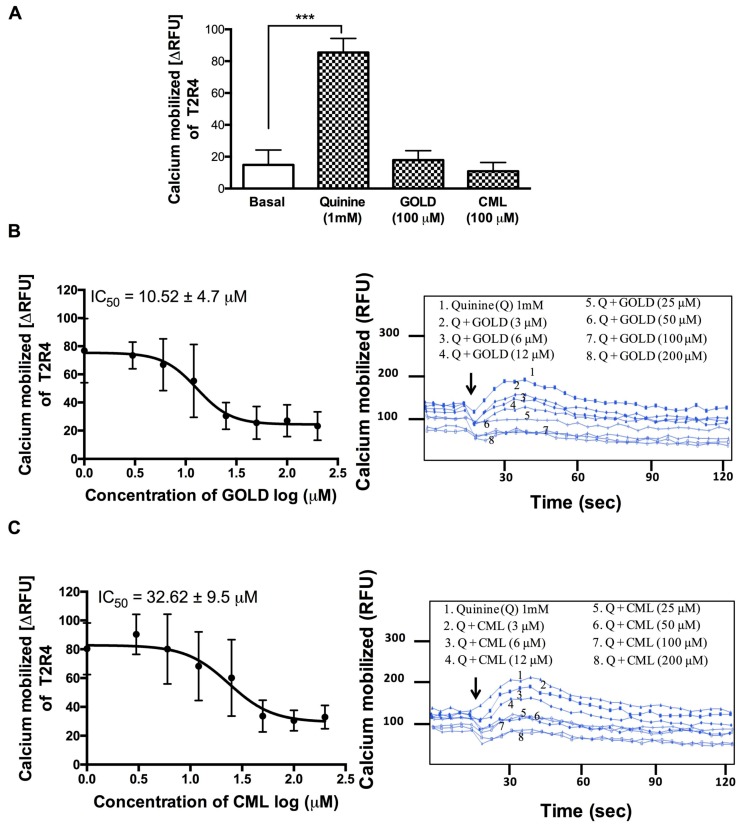
Calcium mobilization assays on T2R4 treated with AGE compounds. (**A**) HEK293T cells stably expressing T2R4 were treated with the AGE compounds, glyoxal-derived lysine dimer (GOLD) and carboxy methyl lysine (CML), at 100 μM. Assay buffer (basal) and quinine (1 mM) were included as negative and positive controls, respectively. One-way ANOVA with Tukey’s multiple comparison post hoc test was performed to determine the statistical significance compared to the basal response. *** *p* < 0.001. The data was from three independent experiments in triplicates with error bars representing the standard deviation. (**B**) and (**C**). Concentration dependent responses of T2R4 expressing cells stimulated with 3–200 μM of GOLD or CML in the presence of quinine (1 mM). Calcium mobilization reduced with the increase in GOLD and CML concentration, with an IC_50_ of 10.52 ± 4.7 μM and 32.62 ± 9.5 μM, respectively (± standard deviation). Representative calcium traces of T2R4 expressed in HEK293T cells stimulated with quinine (Q), Q + GOLD, and Q + CML. Arrows indicate the time point (20 s) at which the ligands were added.

We used T2R4 expressing HEK293T stable cells to evaluate the effects of GOLD and CML on calcium mobilization. GOLD and CML (100 μM) alone did not cause a significant increase in calcium mobilization (Figure 1A). Subsequently, we tested the GOLD and CML for blocking ability in the presence of quinine at 1 mM (EC_50_ for T2R4). To calculate their IC_50_ values, we treated the T2R4 cells with GOLD and CML at a concentration range of 3 μM to 200 μM against a fixed concentration of quinine (1 mM). GOLD showed a significant ability to reduce the calcium levels generated by quinine activated T2R4 in a concentration dependent manner with an IC_50_ 10.52 ± 4 μM (Figure 1B). On the other hand, CML exhibited a moderate level of inhibition of quinine-stimulated calcium mobilization with an IC_50_ 32.62. ± 9 μM (Figure 1C). Together, the results suggested GOLD as a potent antagonist, whilst CML was a weak antagonist of quinine activated T2R4.

### 3.3. Analysis of the Binding Pocket for AGEs in T2R4

**Figure 2:**
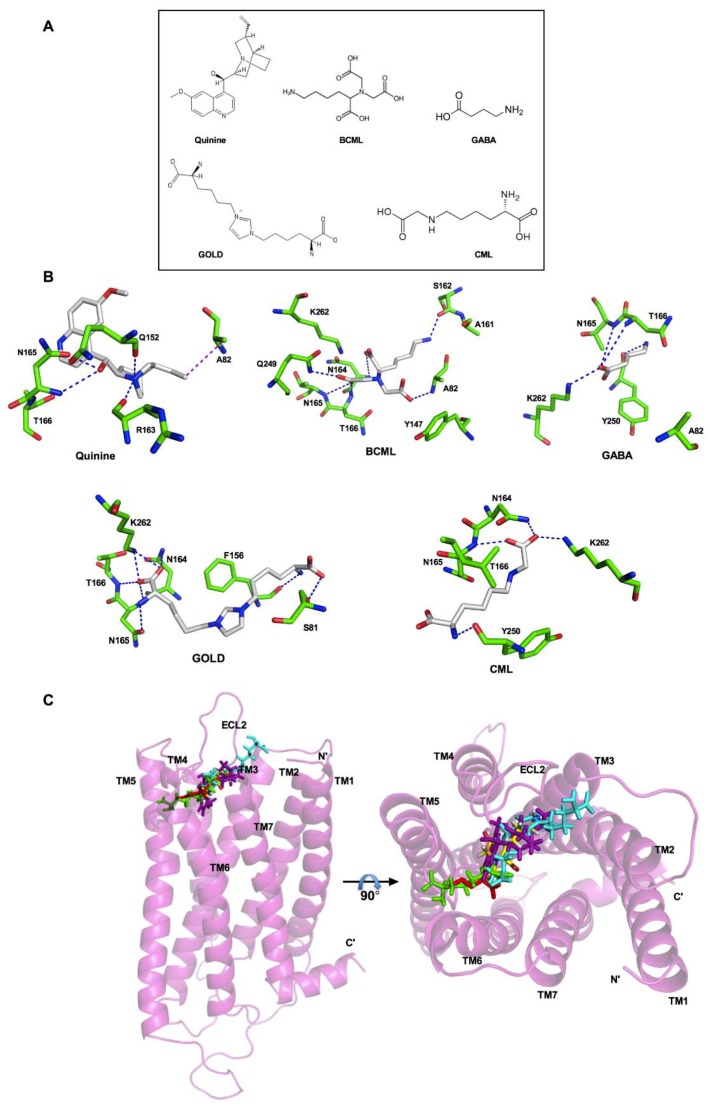
Model of T2R4 bound to GOLD and CML, and a comparison of their binding modes. (**A**) 2D structures of Quinine, BCML, GABA, GOLD, and CML. (**B**) 3D representation of the predicted interactions (dashes) of Quinine, BCML, GABA, GOLD, and CML with the residues (sticks) in the binding pocket of T2R4. The essential interactions with T2R4 are conserved in all the ligands. In contrast to BCML, GOLD and CML show interactions with K262 and not with A82, which is similar to GABA interactions. (**C**) The side view and top view of the superimposed homology models of T2R4 docked with CML (green), GOLD (cyan), BCML (yellow), GABA (red), and Quinine (magenta). The extracellular loop 2 (ECL2) of T2R4 is in close proximity to the docked compounds. TM: transmembrane helix.

For docking and analyses of the binding pocket of AGEs in T2R4, we used an experimentally validated 3D model of T2R4. Previously, the binding pocket of this model was extensively studied for various ligands, including quinine, bacterial stimulants, antibiotics, as well as T2R4 blockers such as GABA and BCML [12,41,42]. To compare the binding interactions of GOLD and CML with the interactions validated by previous mutagenesis experiments, we included quinine, BCML, and GABA as controls (Figure 2A). We docked these three compounds along with GOLD and CML into the previously validated T2R4 model as described in the materials and methods section. The residues within the 4 Å region were selected to analyze the binding interactions with each compound (Figure 2B). Quinine, BCML, and GABA showed similar interactions with T2R4 as observed in the previous studies (Figure 2B) [12,41]. These included the essential interactions with N165 and T166. In addition, we observed the signature interactions of the N1 amino group of A82 with the carboxyl group of BCML, as well as the ε-amino group of K262 with the O1 carboxyl group of GABA. This corroboration with previous studies validated the T2R4 model to analyze the predicted interactions with AGEs. Not surprisingly, GOLD and CML achieved polar interactions with the previously identified essential amino acids N164, N165, and T166 present in ECL2. However, in contrast to BCML both GOLD and CML achieved interactions with the ε-amino group of K262, and not with A82, which was similar to the interactions observed with GABA. There were also compound specific residues that were involved in polar interactions. GOLD interacted with S81 in TM3 and F156 located near the TM4-ECL2 interface, whilst CML interacted with Y250 near the TM6-ECL3 interface. Furthermore, depending on the complexity of structure, the compounds docked at different regions in the binding pocket (Figure 2C). Quinine (magenta) occupied the central region deep into the core, probably influencing the helical bundle rearrangement to attain an active conformation. On the other hand, CML (green) docked slightly deeper near to TM5 and TM6, compared to GOLD (cyan), which seemed to bind towards the extracellular side. Furthermore, CML (green) was found to occupy the region similar to GABA (red).

### 3.4. Effect of GOLD and CML on the Activation of T2R14 and T2R20

**Figure 3:**
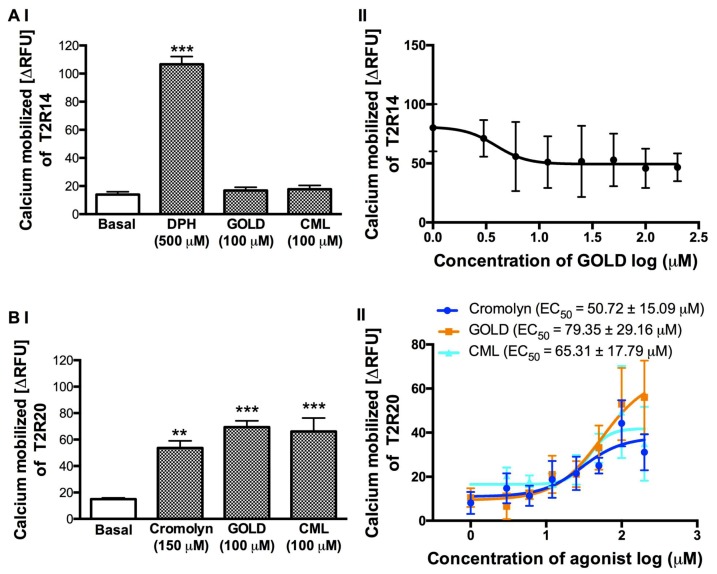
Calcium mobilization assay of T2R14 and T2R20 treated with AGE compounds. We treated the (**A**) **I** and (**B**) **I** HEK293T cells that stably express T2R14 or T2R20 with the AGE compounds GOLD and CML at 100 μM each. Assay buffer (basal) and agonists DPH (500 μM) or cromolyn (150 μM) were included as negative and positive controls, respectively. One-way ANOVA with Tukey’s multiple comparison post hoc test was performed to determine the statistical significance compared to the basal response. *** *p* < 0.001, ** *p* < 0.01. The data was from three independent experiments in triplicates with error bars representing the standard deviation. (**A**) **II**. Concentration dependent curve of T2R14 expressing cells stimulated with increasing concentrations of GOLD (3–200 μM) in the presence of DPH (500 μM). (**B**) **II**. Concentration dependent curves of T2R20 expressing cells stimulated with increasing concentrations of cromolyn (blue), GOLD (orange), and CML (cyan) ranging from 3–200 μM. The calculated EC_50_ for cromolyn, GOLD, and CML are 50.72 ± 15.09 μM, 79.35 ± 29.16 μM, and 65.31 ± 17.79 μM, respectively (± standard deviation).

To test the activity of GOLD and CML on T2R14 and T2R20, which are highly expressed in a number of human tissues, we treated the stable cells expressing these T2Rs in a similar manner to the T2R4 cells. We selected the well-characterized agonists, DPH for T2R14 and cromolyn for T2R20, respectively (Figure 3). GOLD and CML (100 μM) did not show any calcium response with T2R14 cells (Figure 3AI). To determine the IC_50_ of GOLD against DPH activated T2R14, we treated the cells with GOLD at a concentration range of 3–200 μM with DPH at 500 μM. However, although the data showed a reduction of DPH-stimulated calcium levels at higher concentrations of GOLD, the non-linear regression analysis for inhibition did not generate a reverse sigmoid curve to determine the IC_50_ value (Figure 3AII). CML produced no significant effect in suppressing the DPH activation of T2R14 (data not shown).

In contrast to the response with T2R4 and T2R14, GOLD (100 μM) and CML (100 μM) activated T2R20 (Figure 3BI). Therefore, to determine the EC_50_ values, we treated the T2R20 cells with cromolyn, GOLD, and CML in a concentration dependent manner (3–200 μM). The T2R20 agonist cromolyn showed an EC_50_ of 50.72 ± 15.09 μM similar to our previous study [41]. GOLD and CML activated T2R20 with EC_50_ of 79.35 ± 29.16 μM and 65.31 ± 17.79 μM, respectively (Figure 3BII). Together, these results suggested that GOLD and CML exhibited T2R specific activity to inhibit (T2R4, T2R14) or activate (T2R20) the receptors.

## 4. Discussion

Recently, enzymatic protein hydrolysates from beef and hens have been shown to interact with T2Rs [29,30]. Results from the beef study showed that the peptides present in alcalase hydrolysate and chymotrypsin hydrolysate fractions lowered the amount of calcium mobilized by quinine whilst other enzymatic hydrolysates showed minimal effects. In another report, secondary metabolites such as plant-based polyphenols were shown to produce combinatorial activation patterns of TAS2Rs [52]. Considering the wide range of food-derived compounds that interact with T2Rs, it is worthwhile looking into AGEs-T2Rs interactions.

BCML, a synthetic derivative of an AGE compound (CML), can act as a potent T2R4 antagonist [12]. This knowledge led us to further analyze various AGE compounds to identify novel T2R ligands. Out of all the compounds docked with T2R4, CML and GOLD were amongst the top ranked and based on their commercial availability, as well as solubility, we tested them in this work. We treated the T2R4 with both compounds and no significant response (calcium mobilization) was observed when compared to that of quinine. Compounds showing no T2R activation indicate that they might act as antagonists. Thus, we performed a competitive treatment in the presence of quinine on the T2R4-expressing cells. Based on the predicted binding affinity, CML showed a higher affinity than GOLD and this compound was structurally similar to BCML. However, in contrast to BCML (IC_50_ = 60 nM), CML showed very weak inhibition of T2R4 activation even at a high concentration. It has been shown that A82 in T2R4 is an important residue for BCML binding and its effect on quinine-dependent activation [12]. However, in this study, A82 was significantly away from the binding pocket preventing its involvement in interacting with CML, which was involved in interactions with Y250 and K262 apart from the essential amino acids in the extracellular loop two (ECL2). This was similar to the binding interactions of another T2R4 antagonist, GABA, which was previously shown through mutational studies to interact with K262 and not with A82 [12]. Although BCML and CML are structurally similar, the additional carboxyl group in BCML might increase the complexity of the compound, which enabled interaction with residues other than the residues involved with CML. Hence, the two compounds had different affinities towards T2R4. Changes in the potency of similar compounds upon rearranging the structural moieties is not a new aspect in T2R studies. Previously, it was shown that the modifications in T2R14 ligands mefenamic acid and diclofenac resulted in noticeable changes in receptor interaction [53]. A similar phenomenon was also observed in a recent study on phenol compounds interacting with T2R5 and T2R7 [52].

Interestingly, GOLD exhibited a significant blocking effect on T2R4 following an increase in the concentration, with an IC_50_ of 10.52 ± 4.7 μM. GOLD is a bulky compound with two lysine side chains linked to the glyoxal ring. The functional groups in both lysine side chains are involved in the interactions with multiple residues. These observations suggest that the complexity of compounds and the type interactions determine their activity on the receptor. GOLD, a glyoxal derivative, exhibits additional specific interactions with S81 and F156 in T2R4, whilst CML interacts with Y250 (Figure 2B). The docking analysis obtained a stronger predicted receptor-ligand affinity (*K_d_*) value with 46 nM for CML, compared to 110 nM for GOLD (Table 1). However, the results from the calcium mobilization assays indicated that GOLD was a strong blocker of T2R4 than CML. Moreover, the *K_d_* values obtained from the scoring functions in the molecular modeling were predictions of affinity and not efficacy (EC_50_). In our experimental cell based assays, we determined only the efficacy for ligand-T2R-calcium signaling and not affinity. There are various factors influencing receptor efficacy that cannot be captured using the modeling algorithms. These include complex receptor activation mechanisms, the role of membrane lipid composition, biased agonism, and G-protein independent downstream signaling. Experiments performed with labeled bitter compounds would be useful to determine the binding affinities.

The docking analysis provided insights into the binding orientation of these compounds during interactions with T2R4. It appears that complexity of the compounds might play a role in binding and modulating T2R4 function. Apart from the agonist quinine that was bound in the central region of the T2R4 binding pocket covering the TM helices and ECLs, the CML was bound slightly deeper towards TM5 and TM6 and away from ECL2. GOLD being a more complex compound was bound in the center of the cavity extending slightly towards the extracellular part, mostly interacting with ECL2 residues. CML structure is simpler than GOLD and this might be a factor that determined the greater antagonistic effect of GOLD compared to CML against T2R4 activation by quinine. A similar phenomenon with BCML, which is a known antagonist of T2R4, also supports the inference that a complex structure with reactive functional groups might hold the receptor rigid and prevent it from attaining an active conformation in competition with quinine [12]. From our previous studies using multiple compounds, including both agonists and antagonists, we identified crucial amino acids that were conserved to interact with T2R4 ligands irrespective of their functionality as agonist or antagonist [12,42]. Here in this study, we observed that the residues involved in interactions with both GOLD and CML were the conserved amino acids that were crucial to interact with quinine. Therefore, in this scenario, we did not deem it worthwhile to perform site-directed mutagenesis experiments, as the mutations would not differentiate the effects of antagonists on the response of the agonist.

Meanwhile, further analysis on the effects of GOLD and CML on T2R14 and T2R20 activation showed different results. Both the AGEs showed similar effects on T2R14 as observed with T2R4. Although at higher concentrations the DPH response was reduced, there was no dose-dependent inhibition observed with GOLD. In contrast, both AGEs activated T2R20 in a concentration-dependent manner. Our results were similar to a previous study, which showed that a single compound could act as an activator and a blocker of different T2Rs. For example, cyclamate blocked the receptors activated by saccharin, and in turn, saccharin exhibited a blocking effect on T2Rs activated by cyclamate [11]. These results indicated the potential versatile effects of AGEs against different proteins under physiological conditions, e.g., AGE products can down-regulate the gap junctions in human hepatoma cells [54]. Moreover, these compounds had no universal effect as either agonist or antagonist on the tested T2Rs. A huge diversity is present in the extracellular regions of T2Rs, facilitating their versatility to recognize a wide range of compounds. Thus, the challenge remains to identify a universal bitter blocker. The amino acid multiple sequence alignment between these three T2Rs, which was performed in our previous study, indicated that T2R4 shares <26% similarity with T2R14 and T2R20, whilst the latter both share a 42% similarity. However, only a few important residues in the ECL2 region of T2R4 and T2R14 were identified as conserved [41]. Further docking analysis in T2R14 and T2R20 would give more insights into the binding sites of tested compounds and compare the conserved residues among them.

## 5. Conclusions

Several studies have shown that T2Rs are expressed in extra-oral tissues and they play a vital role in health and diseases like diabetes, cancer, asthma, and inflammation. It seems that T2Rs are important in defense mechanisms and they play a beneficiary role in the physiology. Therefore, taken together, the extra-oral functions of T2Rs and the demonstrated involvement of AGE products in pathological conditions, such as diabetes, cancer, and inflammation, it is possible that AGEs may also regulate the cellular functions through interactions with the T2Rs [23,26,55,56]. Further studies are necessary on the above physiological aspects to investigate the role and mechanism of AGE regulation on T2R signaling. Further studies are required to analyze AGEs in processed food extracts, and sensory panel analysis to determine bitter taste-blocking properties.

## Figures and Tables

**Table 1:** Predicted binding affinities of advance glycation end-products (AGEs). CID: PubChem; CSID: ChemSpider; CHEBI: Chemical Entities of Biological Interest (EMBL-EBI), NA: Not Available.

AGE Compound	Chemical Identifier	Predicted Binding Affinity, –log (*K_d_*)	Predicted *K_d_* (μM)
Pentosidine	CID 119593	7.71	0.02
**Nε-Carboxymethyl-lysine (CML)**	CID 123800	7.34	0.04
Tetrahydropyrimidine (THP)	CID 5231957	7.3	0.05
3-deoxyglucosone-derived lysine dimer (DOLD)	NA	7.13	0.07
Nε-Carboxyethyl Lysine (CEL)	CID 23400779	7.04	0.09
**Glyoxal-derived lysine dimer (GOLD)**	CHEBI:59965	6.96	0.11
Argpyrimidine	CID 17750123	6.81	0.15
Methyl glyoxal hydroimidazolone 1(MG-H1)	NA	6.77	0.17
Methyl glyoxal-derived lysine dimer (MOLD)	NA	6.6	0.25
3-deoxyglucosone hydroimidazolone 3 (3DG-H3)	NA	6.51	0.31
Fructosyl Lysine	CID 123708	6.47	0.34
Pyrraline	CID 122228	6.22	0.60
Nε-carboxymethyl-hydroxylysine (CMhL)	CID 125438	6.21	0.62
Glucosepane	CSID 26333276	5.92	1.20
3-deoxyglucosone hydroimidazolone 2 (3DG-H2)	NA	5.78	1.66
ImidazoloneA	CSID 9993693	5.72	1.90
1-Alkyl-2-formyl-3,4-glycosyl-pyrrole (AFGP)	NA	5.66	2.19
Methyl glyoxal hydroimidazolone 2 (MG-H2)	NA	5.62	2.40
ImidazoloneB	CSID 9993693	5.29	5.13
3-deoxyglucosone hydroimidazolone 1 (3DG-H1)	NA	4.77	16.9
Glyoxal-derived hydroimidazolone (G-H)	NA	4.7	19.9
Methyl glyoxal hydroimidazolone 3 (MG-H3)	NA	4.19	64.5

## Abbreviations

AGE	advanced glycation end-product
BCML	*N*α,*N*α-bis(carboxymethyl)-l-lysine
CML	Nε-carboxymethyl-l-lysine
DPH	diphenhydramine
GOLD	glyoxal-derived lysine dimer
HEK293T	human embryonic kidney cells
RAGE	receptor for advanced glycation end-products
RFU	relative fluorescence units
T2Rs	bitter taste receptors
